# Hypogonadism induced by surgical stress and brain trauma is reversed by human chorionic gonadotropin in male rats: A potential therapy for surgical and TBI‐induced hypogonadism?

**DOI:** 10.1002/edm2.239

**Published:** 2021-03-18

**Authors:** Rastafa I. Geddes, Amita Kapoor, Kentaro Hayashi, Ryan Rauh, Marlyse Wehber, Quinn Bongers, Alex D. Jansen, Icelle M. Anderson, Gabrielle Farquhar, Sivan Vadakkadath‐Meethal, Toni E. Ziegler, Craig S. Atwood

**Affiliations:** ^1^ Division of Geriatrics and Gerontology Department of Medicine University of Wisconsin‐Madison School of Medicine and Public Health Madison WI USA; ^2^ Assay Services Unit and Institute for Clinical and Translational Research Core Laboratory National Primate Research Center University of Wisconsin‐Madison Madison WI USA; ^3^ Geriatric Research, Education and Clinical Center Veterans Administration Hospital Madison WI USA; ^4^ School of Exercise, Biomedical and Health Sciences Edith Cowan University Joondalup Australia

**Keywords:** human chorionic gonadotropin, hypoadrenalism, hypogonadism, RU‐486, testosterone, traumatic brain injury

## Abstract

**Introduction:**

Hypogonadotropic hypogonadism (HH) is an almost universal, yet underappreciated, endocrinological complication of traumatic brain injury (TBI). The goal of this study was to determine whether the developmental hormone human chorionic gonadotropin (hCG) treatment could reverse HH induced by a TBI.

**Methods:**

Plasma samples were collected at post‐surgery/post‐injury (PSD/PID) days ‐10, 1, 11, 19 and 29 from male Sprague‐Dawley rats (5‐ to 6‐month‐old) that had undergone a Sham surgery (craniectomy alone) or CCI injury (craniectomy + bilateral moderate‐to‐severe CCI injury) and treatment with saline or hCG (400 IU/kg; i.m.) every other day.

**Results:**

Both Sham and CCI injury significantly decreased circulating testosterone (T), 11‐deoxycorticosterone (11‐DOC) and corticosterone concentrations to a similar extent (79.1% vs. 80.0%; 46.6% vs. 48.4%; 56.2% vs. 32.5%; respectively) by PSD/PID 1. hCG treatment  returned circulating T to baseline concentrations by PSD/PID 1 (8.9 ± 1.5 ng/ml and 8.3 ± 1.9 ng/ml; respectively) and was maintained through PSD/PID 29. hCG treatment significantly, but transiently, increased circulating progesterone (P_4_) ~3‐fold (30.2 ± 10.5 ng/ml and 24.2 ± 5.8 ng/ml) above that of baseline concentrations on PSD 1 and PID 1, respectively. hCG treatment did not reverse hypoadrenalism following either procedure.

**Conclusions:**

Together, these data indicate that (1) craniectomy is sufficient to induce persistent hypogonadism and hypoadrenalism, (2) hCG can reverse hypogonadism induced by a craniectomy or craniectomy +CCI injury, suggesting that (3) craniectomy and CCI injury induce a persistent hypogonadism by decreasing hypothalamic and/or pituitary function rather than testicular function in male rats. The potential role of hCG as a cheap, safe and readily available treatment for reversing surgery or TBI‐induced hypogonadism is discussed.

## Graphical abstract

Traumatic brain injury (TBI) is a major public health problem that induces hypogonadism and exacerbates the length of recovery. We find that craniectomy or TBI in male rats induces a persistent hypogonadism that can be reversed using the developmental hormone human chorionic gonadotropin (hCG). hCG is a cheap and readily available FDA‐approved drug that could be a safe treatment for reversing hypogonadism and improving recovery from brain trauma.

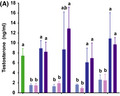

## 1 INTRODUCTION

Traumatic brain injury (TBI) is a major public health problem^1^ due to the relatively high incidence rate (106 per 100,000 globally),^2^ and the lack of effective treatments. The incidence of TBI in males is 3 times that of females, but normalizes to 1:1 by age 65.^3^ The consequences of a TBI can include functional (eg decreased cognitive performance), psychopathological (eg post‐traumatic stress disorder), neuroanatomical (eg cystic infarcts, neurodegeneration) and biochemical (eg inflammation) changes.

An underappreciated endocrinological complication of TBI is hypogonadotropic hypogonadism (HH). TBI can markedly suppress pituitary gonadotropin secretion and gonadal sex steroid production.^4^, ^5^, ^6^, ^7^, ^8^, ^9^, ^10^, ^11^, ^12^, ^13^, ^14^, ^15^, ^16^ Such hypothalamic‐pituitary‐gonadal (HPG) axis hormones have well‐described roles in the formation and maintenance of brain structure and cognitive function (reviewed in Hara et al.^17^). Our early studies utilizing human embryonic stem cells (hESC) as a model of early embryogenesis^18^ identified human chorionic gonadotropin (hCG), a trophoblastic gonadotropin hormone, as modulating the expression and processing of the amyloid‐β precursor protein (AβPP^19^, ^20^). Although this protein has well‐defined neurogenic properties,^21^, ^22^, ^23^, ^24^, ^25^, ^26^ our data suggested AβPP also had important developmental functions during early human embryogenesis (prior to the formation of neural precursor cells). We subsequently determined that hCG signals via the luteinizing hormone/chorionic gonadotropin receptor (LHCGR) on hESC to promote the growth and development of the pre‐implantation embryo, including the formation of the 3 germ layers in the morula and its development into a blastocyst.^27^, ^28^ hCG mediated these effects via the upregulation of steroidogenesis (P_4_ synthesis); P_4_ signalling was found to be obligatory for both embryoid body (aka morula) and neuroectodermal rosette (aka primitive neural tube) formation.

In addition to hCG's role in early embryonic and brain development,^27^ hCG and its adult homolog luteinizing hormone (LH) promote neuronal proliferation,^29^ while hCG and sex steroids regulate adult neuronal differentiation (ie neuritogenesis, spine density, synaptogenesis).^30^, ^31^ Conversely, age‐related or age‐induced reproductive *endocrine dyscrasia* has a negative impact on cognitive function,^32^ an effect that can be slowed or halted by the partial rebalancing of the HPG axis with sex steroid supplementation^33^, ^34^ or gonadotropin‐releasing hormone (GnRH) superagonist and antagonist treatment.^35^ HH could therefore greatly compromise cognitive performance and neuroregeneration following a TBI. Importantly, hCG has been shown to improve functional recovery following spinal cord injury in rats,^30^, ^36^, ^37^ and decrease ischaemic brain injury in adult and neonatal rodent models of stroke.^38^, ^39^, ^40^ Aside from these neuroprotectant properties, hCG also promotes neurite sprouting and neuron survival.^40^

TBI‐induced HH is thought to result from damage to the hypothalamus or pituitary^41^, ^42^ and/or stress‐induced cortisol‐mediated suppression of the HPG axis.^43^, ^44^ While prevalence rates for HH vary widely, likely due to the severity of the injury, location and type of injury, time of screening and design of the study, there is increasing consensus that even mild TBIs can induce HH and that severe TBIs induce persistent HH.^6^, ^14^, ^45^, ^46^, ^47^ This silent condition goes mostly undiagnosed and therefore untreated. Based on the pleiotropic properties of hCG, in this study we tested whether hCG treatment could reverse HH induced by a commonly used TBI model (controlled cortical impact; CCI). We find that hCG treatment reverses HH induced by either a craniectomy alone, or a craniectomy and penetrating CCI injury, in adult male rats.

## 2 MATERIALS AND METHODS

### 2.1 Subjects

Male Sprague‐Dawley rats (*n* = 58, 5 to 6 months old) were acquired from Harlan Laboratories Inc. (Madison, WI) and acclimated to the environment over 2 days. Rats were then weighed and handled for no less than 5 min. each for 5 consecutive days and daily thereafter while being housed, fed and maintained on a 12‐h reverse light/dark cycle. The Institutional Animal Care and Use Committee (Animal Component of Research Protocol) at the William S. Middleton Veterans Administration Hospital approved the procedures used in this study, and the research was conducted in an AAALAC‐approved facility. Experimenters were blinded as to the identity of the animals throughout injections, blood collections, and body weight and hormone data analyses.

### 2.2 Surgeries

All surgical procedures were carried out under isoflurane gas anaesthesia (5% for induction; 1.5%–3.0% for maintenance, craniectomy ~15‐ to 30‐min duration; craniectomy +CCI injury ~25‐ to 45‐min duration). An anaesthesia chamber was used for induction, and a nose cone was used for maintenance.

#### 2.2.1 Controlled cortical impact and sham surgeries

Anaesthetized rats were mounted in a Kopf stereotaxic device (Model 900), where the animal's head was held in place by non‐traumatic ear bars and a bite bar. Anaesthesia was maintained by nose cone while the head was shaved and sterilized with 70% ethanol and Betadine™ (Purdue Products L.P.) antiseptic solution. Throughout surgery, anaesthesia levels were monitored closely and were frequently adjusted as needed, based on heart rate, respiration rate and oxygen saturation. A homeothermic blanket control unit (Harvard Apparatus, Holliston, MA) was used to monitor body temperature and to prevent hypothermia throughout surgery.

Under aseptic conditions, the cranium and its bony landmarks including bregma (β) and lambda (λ) were exposed by making a midline incision along the scalp into the skin and fascia covering the skull. A 6‐mm‐diameter craniectomy was centred on the midline at 2.5 mm anterior to β. The cortical impact was made at 2.5 mm anterior to β over the midline of the medial frontal cortex with an Impact One™ Stereotaxic CCI instrument (Leica), using a 5 mm impactor (bit size), travelling at 2.25 m/s (velocity), extending 3 mm below the cortical surface (impact depth) for 100 ms (dwell time). Sham‐injured groups received the same surgical procedures up to and including craniectomy but no CCI injury. After surgery, the rats were placed on a heating pad, monitored closely and upon awakening were tested 30 min later for righting reflex to assess any immediate effects of craniectomy or CCI injury on righting ability, and then returned to their home cages.

### 2.3 Experimental design

Once out of quarantine all rats were weighed and handled for one week. The final body weights at the end of this week were (1) ranked from highest to lowest (as a function of age) and then (2) used to assign each rat to a surgery/treatment group in a counterbalanced manner (ie using the ABBA method).

#### 2.3.1 Experiment 1

Rats were assigned to the following groups: Sham + saline (*n* = 5), Sham + hCG (*n* = 5), CCI + saline (*n* = 8), CCI + hCG (*n* = 8). Baseline blood draws were collected from all animals. Beginning 1 hour after craniectomy or CCI, Pregnyl^®^ (hCG, 400 IU/kg; Merck & Co., Inc.) or saline (0.9% NaCl in deionized H_2_O; equivalent volume to that injected for hCG) was injected intramuscularly every other day for 29 days. Pregnyl^®^ is a highly purified pyrogen‐free preparation obtained from the urine of pregnant females. Each vial contains 10,000 USP units of sterile dried powder with 5 mg monobasic sodium phosphate and 4.4 mg dibasic sodium phosphate that is diluted in solvent containing water, 0.56% sodium chloride and 0.9% benzyl alcohol (https://www.drugs.com/pro/pregnyl.html).

#### 2.3.2 Experiment 2

Rats were assigned to the following groups: Sham + saline + RU‐486 (*n* = 5), Sham + hCG + RU‐486 (*n* = 5), CCI + saline + RU‐486 (*n* = 5), or CCI + hCG + RU‐486 (*n* = 5). RU‐486 (Mifepristone, 100 mg/ml solution, CAS Number 84371‐65‐3; 40 mg/kg in 100% ethanol; Sigma‐Aldrich Corp.) was injected intraperitoneally 15–20 min before every hCG or saline control injection.

### 2.4 Blood collection and hormone analyses

Rats were anaesthetized (between 9:00 a.m.–12:00 noon) and their tails placed in a 200‐ml beaker filled with warm water (≤44°C) for 5 min. The tail was cleaned with 70% alcohol, the minimal amount of the tail tip snipped with a blade, and/or the wound reopened by removal of the scab for subsequent bleeds, and ~1 ml of whole blood was collected directly into EDTA tubes at baseline (post‐injury day (PID)−10) and at PID, 1, 11, 19 and 29. Blood collected did not exceed 1% of body weight every 2‐week period. Animals were injected with Lactate Ringers solution (5 ml) for fluid resuscitation. At the terminal bleed (day 29), blood also was collected via heart puncture. Collected blood was immediately centrifuged at 4000 *g* for 10–20 min. and the plasma aliquoted into Eppendorf tubes for storage at −80°C. Plasma samples were analysed at the Assay Services Laboratory in the Wisconsin National Primate Research Center of the UW‐Madison Institute for Clinical and Translational Research for progesterone (P_4_), testosterone (T), 11‐deoxycorticosterone (11‐DOC) and corticosterone adapted from a method previously described.^48^, ^49^ Briefly, to plasma samples (400 μl) internal standard (200 pg d9‐progesterone and d5‐testosterone and 1 ng d4‐cortisol) was added and the samples were extracted with methyl tert butyl ether. The organic phase was transferred to a clean vial and evaporated to dryness, and then, a second dichloromethane extraction was performed. The organic phase was transferred into a clean test tube and evaporated to dryness and reconstituted in mobile phase. Samples were analysed on a QTRAP 5500 quadruple linear ion trap mass spectrometer (AB Sciex) equipped with an atmospheric pressure chemical ionization source. The system includes two Shimadzu LC20ADXR pumps and a Shimadzu SIL20ACXR autosampler. A sample of 30 μl was injected onto a Phenomenex Kinetex 2.6u C18 100A, 100 × 2.1 mm column (Torrance, CA) for separation using a mobile phase: water with 1% formic acid (Solution A) and acetonitrile with 1% formic acid (Solution B), at a flow rate of 200 μl/min. Quantitative results were recorded as multiple reaction monitoring (MRM) area counts after determination for the response factor for each compound and internal standard. Each steroid had a MRM used for quantitation and 1 or 2 additional MRMs as qualifiers. The linearity was *r* > .818, and the curve fit was linear with 1/*x* weighting. None of the compounds of interest were detected in blank or double blank samples. Inter‐assay coefficients of variation were determined from a pool of rat plasma: T—2.1%, P_4_—11.2%, 11‐DOC—5.3%, corticosterone—8.0%, androstenedione—13.3%.

### 2.5 Statistical analysis

A mixed factorial analysis of variance (ANOVA) for repeated measures was performed on the weight, behavioural, hormonal and gross lesion data (GraphPad Prism, v.7; GraphPad Software, Inc.). Post hoc analyses were performed using the Tukey multiple comparison test. Independent paired *t* tests were also used to compare the differences between baseline (pre‐injury) and post‐injury data when data were normally distributed. Hormone data collected on post‐surgery (PSD) or post‐injury (PID) days were analysed using the R statistical program, V.3.4.1 (R: A language and environment for statistical computing (program), Vienna, Austria: R Foundation for Statistical Computing, 2008), with package ‘rmcorr’ (rmcorr: repeated‐measures correlation (program), R package version 0.2.0, 2017), based on Bland and Altman's^50^, ^51^ statistical technique. Non‐repeated baseline data were analysed using Pearson's correlation with the R program. Statistical significance was established at *p* ≤ .05.

## 3 RESULTS

### 3.1 Craniectomy and controlled cortical impact injury induce hypogonadism and hypoadrenalism

ANOVA indicated a significant main effect of treatment for T (*F*(3,82) = 14.35, *p* < .0001), P_4_ (*F*(3,80) = 11.34, *p* < .0001), 11‐DOC (*F*(3,88) = 9.27, *p* < .0001) and corticosterone (*F*(3,88) = 24.98, *p* < .0001). A main effect of day was found for P_4_ (*F*(3,80) = 5.26, *p* = .0023), but not for T (*F*(3,82) = 1.02, *p* = .386), 11‐DOC (*F*(3,88) = 1.31, *p* = .277) and corticosterone (*F*(3,88) = 0.91, *p* = .440). No main effects of treatment × day interaction were found for T (*F*(9,82) = 0.33, *p* = .964), P_4_ (*F*(9,80) = 1.74, *p* = .095), 11‐DOC (*F*(9,88) = 1.07, *p* = .396) or corticosterone (*F*(9,88) = 0.94, *p* = .498).

**FIGURE 1:**
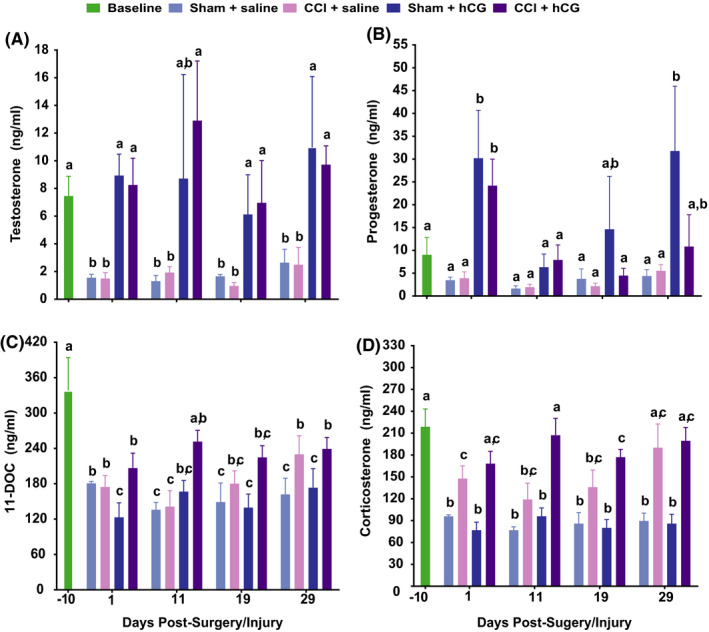
hCG reverses CCI‐induced decreases in circulating testosterone and progesterone. Plasma concentrations (mean ± SEM) of T (A), P_4_ (B), 11‐DOC (C) and corticosterone (D) in ng/ml on PID ‐10, 1, 11, 19 and 29 for the following groups: Sham +vehicle (*n* = 5), Sham +hCG (*n* = 5), CCI +saline (*n* = 8) and CCI +hCG (*n* = 8). Data were analysed using 2‐way repeated‐measures ANOVA; post hoc analyses were performed using the Tukey multiple comparison test (*p* < .05; letters indicate differences between treatment groups and pre‐ and post‐injury days)

Sham surgery (craniectomy + saline group) in male adult rats induced a decline from baseline in the circulating concentrations of T (79.1%; 7.5 ± 1.5 ng/ml to 1.6 ± 0.3 ng/ml; *p* < .05), P_4_ (61.6%; 9.0 ± 3.8 ng/ml to 3.5 ± 0.7 ng/ml; *p* = .061), 11‐DOC (46.6%; 338.3 ± 55.8 ng/ml to 180.7 ± 3.3 ng/ml, *p* < .05) and corticosterone (56.2%; 218.7 ± 24.5 ng/ml to 95.9 ± 2.2 ng/ml, *p* < .05) by PSD1 (Figure 1).^52^ Similar declines in circulating concentrations of T (80.0%, 1.5 ± 0.4 ng/ml, *p* < .01), P_4_ (56.8%, 3.9 ± 1.4 ng/ml; *p* = .065), 11‐DOC (48.4%, 174.5 ± 19.4 ng/ml; *p* < .05) and corticosterone (32.5%, 147.7 ± 17.4 ng/ml; *p* < .05) were observed by PID 1 for CCI‐injured animals (ie craniectomy + CCI + saline group), indicating that Sham surgery alone was sufficient to induce hypogonadotropic hypogonadism (Figure 1A,B) and hypoadrenalism (Figure 1C,D).^52^ Circulating concentrations for all hormones in both Sham surgery and CCI‐injured animals remained at these lower concentrations through PSD/PID 29 (except corticosterone in the CCI group on PID 29, which rose to 190.0 ± 33.0 ng/ml, Figure 1D). Circulating concentrations of androstenedione did not significantly change from baseline (0.58 ± 0.10 ng/ml) in Sham surgery or CCI injury groups (data not shown). These results suggest that sham surgery, and sham surgery plus a bilateral moderate‐to‐severe CCI injury, induces hypogonadism in rats.

### 3.2 hCG reverses craniectomy and CCI‐induced hypogonadism and attenuates hypoadrenalism

hCG treatment of animals that underwent a craniectomy (Sham surgery) or craniectomy plus CCI injury (CCI group) significantly increased circulating concentrations of T and P_4_ back to baseline concentrations by PSD/PID 1 (Figure 1A,B). Unlike P_4_, elevations in T were maintained through PSD/PID 29. While a significant main effect of treatment group for androstenedione concentration also was identified, post hoc analyses determined that androstenedione concentration was only elevated on PID 11 in the CCI +hCG group (4.5 ± 2.1 ng/ml) compared with the CCI +saline group (0.51 ± 0.2 ng/ml, *p* < .03). hCG treatment transiently reduced circulating 11‐DOC in Sham surgery animals (123.1 ± 24.6 ng/ml) when compared to Sham +saline animals (180.7 ± 3.3 ng/ml; *p* < .05) on PID 1, but not on PID 11, 19 and 29 (Figure 1C). hCG treatment had no effect on increasing corticosterone concentrations in Sham animals at any time point, but did increase circulating corticosterone in the CCI animals on PID 1 and 11 (Figure 1D). Together, these results demonstrate that hCG can reverse hypogonadism induced by a craniectomy or a craniectomy +CCI injury, but has lesser effect on reversing hypoadrenalism.

### 3.3 RU‐486 diminishes craniectomy + CCI‐induced hypoadrenalism

ANOVA indicated a significant main effect of treatment for T (*F*(3,71) = 3.76, *p* = .0145), P_4_ (*F*(3,61) = 5.82, *p* = .0011), 11‐DOC (*F*(3,61) = 3.66, *p* = .0171), and corticosterone (*F*(3,61) = 5.41, *p* = .0023). A main effect of day was found for P_4_ (*F*(3,61) = 5.82, *p* = .0015) and corticosterone (*F*(3,61) = 3.38, *p* = .0238), but not for T (*F*(3,71) = 1.86, *p* = .145) or 11‐DOC (*F*(3,61) = 1.80, *p* = .158). No main effects of treatment × day interaction were found for T (*F*(9,71) = 0.33, *p* = .965), P_4_ (*F*(9,61) = 1.09, *p* = .3838), 11‐DOC (*F*(9,61) = 0.54, *p* = .836) or corticosterone (*F*(9,61) = 0.65, *p* = .750).

**FIGURE 2:**
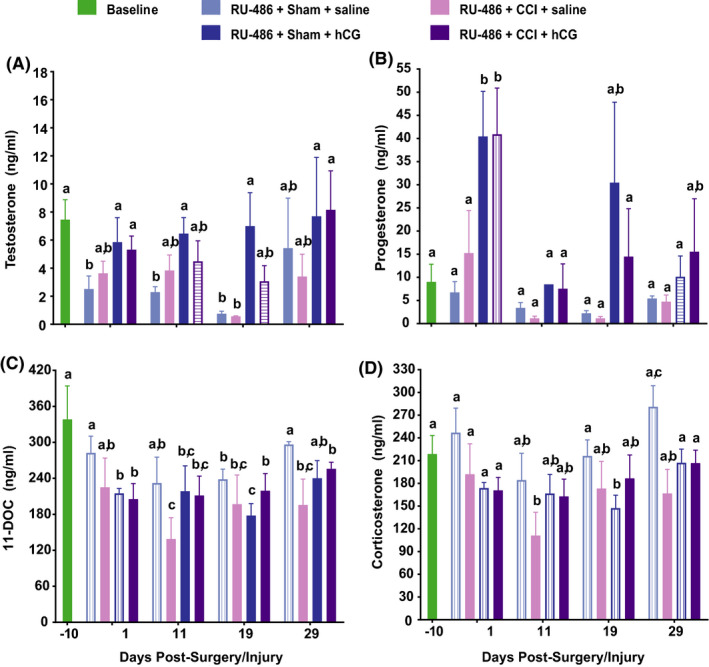
RU‐486 treatment attenuates hCG‐induced reversal of circulating testosterone plasma concentrations. Plasma concentrations (mean ± SEM) of T (A), P_4_ (B), 11‐DOC (C) and corticosterone (D) in ng/ml on PID −10, 1, 11, 19 and 29 for the following groups: RU‐486: Sham +vehicle (*n* = 5), RU‐486: Sham +hCG (*n* = 5), RU‐486: CCI +saline (*n* = 5) and RU‐486: CCI +hCG (*n* = 5). Data were analysed using 2‐way repeated‐measures ANOVA; post hoc analyses were performed using the Tukey multiple comparison test (*p* < .05; letters indicate differences between treatment groups and pre‐ and post‐injury days). Differences between RU‐486‐induced changes in plasma hormones between treatment groups in Figures 1 and 2 are illustrated by (1) vertical lines represent an increase in plasma hormone concentration in RU‐486‐treated animals, and (2) horizontal lines represent a decrease in plasma hormone concentration in RU‐486‐treated animals

Pretreatment of animals with RU‐486, a P_4_ receptor and glucocorticoid receptor antagonist, had little effect on Sham +hCG animals suppressing only P_4_ concentration on PID 29 (8.5 ± 4.5 ng/ml vs. 31.8 ± 14.2 ng/ml, *p* < .05; compare Figures 1B and 2B). In CCI‐injured animals, RU‐486 pretreatment increased P_4_ concentrations on PID 1 (40.9 ± 10.0 ng/ml vs. 24.2 ± 5.8 ng/ml, *p* < .05), and suppressed T concentrations on PID 11 (4.5 ± 1.5 ng/ml vs. 12.9 ± 4.3 ng/ml, *p* < .05) and PID 19 (3.1 ± 1.1 ng/ml vs. 9.7 ± 1.4 ng/ml, *p* < .05; Figure 2A,B). RU‐486 pretreatment had more significant effects on 11‐DOC and corticosterone, preventing the Sham + saline treatment‐induced decrease in 11‐DOC through PID 29 (Figure 2C) and preventing in Sham +hCG rats the decrease in 11‐DOC at PID 1 (282.4 ± 28.0 ng/ml vs. 123.1 ± 24.6 ng/ml, *p* < .05). RU486 pretreatment prevented Sham surgery‐induced decreases in corticosterone through PID 29 in both saline‐ and hCG‐treated animals (except on PID 19 in the Sham + hCG group; Figure 2D). RU‐486 pretreatment had no significant effects on circulating 11‐DOC and corticosterone in CCI‐injured animals (Figure 2C,D).

### 3.4 Relationships between circulating steroid concentrations before and after sham surgery, CCI injury and hCG treatment

Correlation analyses demonstrated strong positive correlations in baseline plasma samples between P_4_ with androstenedione (*r* = .84, *p *< .01), androstenedione with its metabolite T (*r* = .82, *p* < .01) and with corticosterone and its precursor 11‐DOC (*r* = .89, *p* < .001; Table 1). Sham injury obviated the significant correlations between sex steroids, but not corticosterone and its precursor 11‐DOC (r = 0.98, *p* < .001, Table 2). hCG treatment of Sham animals was sufficient in restoring the strong positive relationship between T and androstenedione (*r* = .76, *p* < .05), but not between P_4_ with androstenedione. hCG treatment induced two additional positive correlations between androstenedione with 11‐DOC (*r* = .84, *p* < .01) and androstenedione with corticosterone (*r* = .72, *p* < .05; Table 2). These results suggest that Sham surgery alone is sufficient to disrupt the relationship between sex steroid metabolism, a relationship that is partially reversed with hCG treatment.

**TABLE 1:** The relationship between the concentrations of plasma steroids in rats

		All groups and time points (PSD/PID 1, 11, 19 and 29)				
		T	P_4_	Androstenedione	11‐DOC	Corticosterone
Baseline samples (PSD/PID ‐10)	T		0.13 104	0.68*** 92	0.22 104	0.15 104
	P_4_	0.41 10		0.28* 92	0.29* 104	0.25* 104
	Androstenedione	0.82** 10	0.84** 10		0.33** 92	0.19 108
	11‐DOC	0.40 10	−0.15 10	0.06 10		0.90*** 104
	Corticosterone	0.27 10	−0.28 10	−0.11 10	0.89*** 10	

Note

The top figure in each square is the coefficient of determination (*r* ^2^), and the bottom figure is the number of pairs that were analysed. The results comprising the bottom left triangle are for rats at baseline and the top right triangle are for rats from all time points: post‐surgery days (PSD)/post‐injury days (PID) 1, 11, 19 and 29 for all treatment groups (*p < .05, **p < .01, ***p < .001).

**TABLE 2:** The relationship between the concentrations of plasma steroids in Sham surgery rats treated with or without hCG

		Sham + Saline (PSD 1, 11, 19 and 29)				
		T	P_4_	Androstenedione	11‐DOC	Corticosterone
Sham + hCG (PSD 1, 11, 19 and 29)	T		0.29 20	0.04 20	<0.01 20	0.08 20
	P_4_	0.05 20		0.11 20	0.84*** 20	0.87*** 20
	Androstenedione	0.76* 20	0.29 14		0.34 20	0.34 20
	11‐DOC	0.44 20	0. 27 20	0.84** 14		0.98*** 20
	Corticosterone	0.25 14	0.24 20	0.72* 14	0.94*** 20	

Note

The top figure in each square is the coefficient of determination (*r* ^2^), and the bottom figure is the number of pairs that were analysed. The results comprising the bottom left triangle are for Sham surgery rats treated with hCG and the top right triangle are for Sham surgery rats treated with saline, from post‐surgery days (PSD) 1, 11, 19 and 29 (*p < .05, **p < .01, ***p < .001).

CCI injury, like sham injury, resulted in positive relationships between P_4_ with corticosterone (*r* = .58, *p* < .01) and 11‐DOC (*r* = .68, *p* < .001), between corticosterone and its precursor 11‐DOC (*r* = .91, *p* < .001; Table 3), as well as the loss of significant correlations between sex steroids. Unlike sham injury, CCI injury resulted in a strong correlation between androstenedione with its metabolite T (*r* = .97, *p* < .001). Like Sham animals, hCG treatment of CCI animals restored the positive relationship between T and androstenedione (*r* = .64, *p* < .001), while the positive correlation between corticosterone and 11‐DOC (*r* = .93, *p* < .001) was maintained. Together, these results suggest that like Sham surgery, CCI injury disrupts sex steroid metabolism, while hCG treatment partially restores sex steroid metabolism.

**TABLE 3:** The relationship between the concentrations of plasma steroids in CCI‐injured rats treated with or without hCG

		CCI + Saline (PID 1, 11, 19 and 29)				
		T	P_4_	Androstenedione	11‐DOC	Corticosterone
CCI + hCG (PID 1, 11, 19 and 29)	T		0.04 32	0.97*** 32	−0.06 32	−0.10 32
	P_4_	0.27 32		0.08 32	0.67*** 32	0.58** 32
	Androstenedione	0.64*** 32	0.30 32		−0.03 32	−0. 08 32
	11‐DOC	0.26 32	0.30 32	0.20 32		0.91*** 32
	Corticosterone	0.32 32	0.31 32	0.20 32	0.93*** 32	

Note

The top figure in each square is the coefficient of determination (*r* ^2^), and the bottom figure is the number of pairs that were analysed. The results comprising the bottom left triangle are for CCI injury rats treated with hCG and the top right triangle are for CCI injury rats treated with saline, from post‐injury days (PID) 1, 11, 19 and 29 (*p < .05, **p < .01, ***p < .001).

## 4 DISCUSSION

We demonstrate for the first time that intraperitoneal injection of hCG is effective in reversing hypogonadism (Figure 1A,B) and attenuating hypoadrenalism (Figure 1C,D) following a craniectomy or craniectomy +CCI injury in young adult male rats. Both craniectomy and craniectomy +CCI injury promoted corticosteroid production in favour of sex steroid (P_4_) production, a relationship that was reversed with hCG treatment (Figure 1; Tables 1, 2, 3). hCG's ability to increase sex steroid plasma concentrations following a craniectomy and following a moderate‐to‐severe brain injury supports its potential as a treatment for TBI‐induced hypogonadism.

It is important to note that while hCG has potential to reverse hypogonadism and promote neurogenesis and cognitive recovery, an increase in circulating hCG/LH concentrations as a result of ovariectomy^53^, ^54^, ^55^, ^56^, ^57^, ^58^ or treatment^59^, ^60^ has been shown to impair cognition in rodents, while lowering LH or blocking LHCGR signalling is protective of memory in rodents^53^, ^54^, ^56^, ^57^, ^58^, ^60^, ^61^, ^62^, ^63^ and humans.^35^ Conversely, one study has demonstrated that intracerebroventricular hCG delivery after OVX rescued dendritic spine density and spatial memory.^64^ The general negative impact of LH/hCG on cognitive performance appears to be dependent upon the ratio of gonadotropins to sex steroids since situations where gonadotropins and sex steroids are in balance such as during the adult reproductive period^65^ are periods of normal cognitive performance and do not involve dyotic signalling.^32^, ^33^, ^34^ This is illustrated by the findings that interventions that reverse dyotic signalling such as sex steroid supplementation of ovariectomized animals (see references above), GnRH agonist suppression of gonadotropins in post‐menopausal women^35^ and caloric restriction (eg Ref. 66), either reverse or halt cognitive decline. Therefore, one might predict that functioning gonads are essential for hCG to promote cognitive recovery from a TBI, as we have found in intact male rats^67^ (unpublished data). Thus, hCG treatment might be expected to be most beneficial in pre‐menopausal and pre‐andropausal individuals, while those further along the post‐reproduction spectrum might benefit most from a combination therapy of hCG supplemented with appropriate sex steroids.

### 4.1 Causes of craniectomy and CCI injury induced hypogonadism and hypoadrenalism

The induction of hypogonadism and hypoadrenalism in young male rats following a craniectomy and a craniectomy +CCI injury (reduction in plasma concentrations of P_4_, T, 11‐DOC and corticosterone; Figure 1) is consistent with previous reports in rats.^53^, ^68^ Since hCG reversed hypogonadism and diminished hypoadrenalism in Sprague‐Dawley rats (Figures 1 and 2, Tables 1, 2, 3), our results suggest isoflurane and/or surgical trauma/stress to the HP are impacting the long‐term hypothalamic release of GnRH or the pituitary release of gonadotropins rather than the production of steroids by the testes (or adrenals). Circulating cortisol concentrations are elevated following neurosurgical procedures in humans,^69^, ^70^, ^71^ but not during anaesthesia (nitrous oxide and halothane, after thiopentone induction). Alternatively, or in conjunction, isoflurane anaesthesia administered during the craniectomy may have inhibited hypothalamic and/or pituitary function since it has been reported that isoflurane anaesthesia can dose‐dependently suppress circulating follicle‐stimulating hormone and T concentrations, post‐natal neurogenesis and cognitive performance in adult male Sprague‐Dawley rats.^72^, ^73^, ^74^, ^75^ Anaesthesia‐induced hypogonadism and hypoadrenalism represents another complication of anaesthesia that could impact the recovery from and quality of life for those undergoing anaesthesia for a surgical procedure. Further research is required to delineate whether this effect is attributed to isoflurane on the functioning of the hypothalamus and/or pituitary, a combination of isoflurane and surgical stress or an effect of isoflurane early and of surgical stress later in the maintenance of the hypogonadism over 29 days.

Our data are consistent with early studies in humans demonstrating that TBI could alter hypothalamic morphology and induce hypogonadism and hypothyroidism.^76^, ^77^ However, while our study implicates isoflurane in the induction of hypogonadism and hypopituitarism via suppression of HP function, human studies suggest that TBI‐induced hypogonadism and hypopituitarism is mediated via suppression of HP function by elevated circulating cortisol.^78^, ^79^ Ranganathan et al.^79^ demonstrated that the stress of TBI results in anovulation and central hypothalamic‐pituitary‐ovarian axis suppression, with menstruation resuming among pre‐menopausal women when serum cortisol normalized to luteal phase control levels. It is apparent that TBI‐induced HH, even when limited to the anterior hypothalamus,^13^, ^80^ is a system problem commonly involving both the HPG axis and the HPA axis.^81^ From our study, it is not possible to determine whether the CCI injury had an impact beyond that of craniectomy on promoting hypogonadism or hypoadrenalism, as has been reported for human TBI.^6^, ^7^, ^8^, ^10^, ^14^, ^76^, ^77^, ^78^, ^82^, ^83^, ^84^, ^85^, ^86^, ^87^, ^88^, ^89^, ^90^, ^91^, ^92^, ^93^, ^94^, ^95^, ^96^ These results demonstrate that future studies need to take into account the effects of isoflurane alone in any model of TBI‐induced hypogonadism.

### 4.2 hCG treatment for reversing hypopituitarism

Our results in craniectomized and craniectomized plus CCI‐injured rats demonstrate that post‐surgery and post‐injury male rats retain the capacity to synthesize and secrete T (Figure 1). The reversal of hypothalamic/pituitary function in animals induced by a TBI, craniectomy and/or isoflurane anaesthesia indicates the utility of hCG for reversing hypogonadism and hypoadrenalism in these conditions. hCG treatment comes with the advantage of not only increasing neurotropic hCG/LH, but also increasing the dozens of gonadal sex steroid and protein hormones that regulate normal brain structure and function.^32^

hCG has been shown to increase T production in aged male rats.^97^, ^98^ hCG is a safe, cheap, FDA‐approved treatment for hypogonadism in men (chronically), infertility in men and women, and to promote the descent of testicles in young boys with cryptorchidism.^99^ In this context, hCG treatment has recently been shown to be effective in (1) raising plasma T concentrations in healthy men with chronic spinal cord injury, and this was not significantly different from hCG’s elevation of plasma T in able‐bodied male control subjects,^100^ (2) protecting the rodent adult^37^, ^38^ and neonatal brain from hypoxic‐ischaemic cellular degeneration in vivo and inhibiting glutamate‐dependent excitotoxic or necrotic neuronal cell death in vitro^40^; and (3) increasing ERK phosphorylation, neurite outgrowth and rescuing ovariectomy‐induced spatial memory deficits in C57Bl/6J mice.^64^ In addition, hCG also partially attenuated hypoadrenalism in male rats. Although there are few studies that have assessed the impact of hCG on regulating adrenal steroid production, hCG has been demonstrated to increase follicular fluid concentrations of 11‐DOC, but not corticosterone,^101^ while LHβ overexpressing female mice have enlarged adrenals, increased LHCGR expression and a 14‐fold elevation in serum corticosterone.^102^ In this latter study, the authors proposed that enhanced ovarian oestrogen synthesis causes increased secretion of prolactin, which elevates LHCGR expression in the mouse adrenal cortex, leading to elevated, LH‐dependent, corticosterone production.^102^ Continuous exposure to hCG is, however, known to suppress the expression of LHCGR via the down‐regulation of mRNA (eg Ref. 103, 104). To circumvent the down‐regulation of the receptor, in our study hCG was administered in the form of Pregnyl every other day, as is used clinically.^105^ Since initial phase half‐life of urinary‐derived Pregnyl is between 5.6 and 11 h (https://www.merck.ca/), the 48 h between doses appears sufficient to maintain LHCGR expression, as circulating concentrations of sex steroids (Figure 1A,B) were sustained over the 29‐day experiment.

### 4.3 RU‐486 impact on plasma steroid concentrations

Elevations in circulating corticosterone observed in our study following RU‐486 treatment are consistent with elevations in corticosterone in the male rats^106^ and cortisol, corticotropin or adrenocorticotropic hormone that is observed in human men,^107^, ^108^ women,^108^, ^109^ and non‐human primates (*Macaca fascicularis* ^110^). Blocking glucocorticoid and P_4_ signalling using RU‐486 had little effect on sex steroid changes induced by craniectomy (uninjured) or CCI injury, but significantly diminished the decline in 11‐DOC and corticosterone concentrations in craniectomy (uninjured) but not CCI‐injured animals (Figure 2A–D), indicating that blocking glucocorticoid (and perhaps P_4_) receptor signalling partially prevents the suppression of 11‐DOC and corticosterone (either by limiting stress‐induced suppression of 11‐DOC/corticosterone or by elevating their synthesis).

## 5 LIMITATIONS OF THE STUDY

The suppression of circulating sex steroids by the Sham surgery procedures was unexpected, and the “Sham CCI” group cannot be considered a normal “Control” group in the usual sense of the term. Nonetheless, the fact that stress‐induced HH can be reversed with hCG administration is important from the perspective of a potential treatment option for veterans returning from combat with stress‐ or TBI‐induced HH.^6^, ^14^, ^45^, ^46^, ^47^

## CONFLICT OF INTEREST

The authors declare that they have no competing interests.

## AUTHORS' CONTRIBUTIONS

RIG, KH, RR, MW, QB, ADJ, IMA, GF and CSA performed surgeries, controlled cortical impact injuries and collected blood. AM and TEZ performed blood hormone analyses. RIG, SVM and CSA performed data and statistical analyses. CSA, SVM and RIG conceived the study.

## Data Availability

The data that support the findings of this study are available from the corresponding author upon reasonable request.
